# SABE-YOLO: Structure-Aware and Boundary-Enhanced YOLO for Weld Seam Instance Segmentation

**DOI:** 10.3390/jimaging11080262

**Published:** 2025-08-06

**Authors:** Rui Wen, Wu Xie, Yong Fan, Lanlan Shen

**Affiliations:** 1 Guangxi Key Laboratory of Trusted Software, Guilin University of Electronic Technology, Guilin 541004, China; wenruiguet@126.com; 2 School of Mechanical and Electrical Engineering, Guilin University of Electronic Technology, Guilin 541004, China; 3 School of Computer Science and Information Security, Guilin University of Electronic Technology, Guilin 541004, China; llshen_huahai@126.com

**Keywords:** weld seam recognition, deep learning, instance segmentation, YOLO

## Abstract

Accurate weld seam recognition is essential in automated welding systems, as it directly affects path planning and welding quality. With the rapid advancement of industrial vision, weld seam instance segmentation has emerged as a prominent research focus in both academia and industry. However, existing approaches still face significant challenges in boundary perception and structural representation. Due to the inherently elongated shapes, complex geometries, and blurred edges of weld seams, current segmentation models often struggle to maintain high accuracy in practical applications. To address this issue, a novel structure-aware and boundary-enhanced YOLO (SABE-YOLO) is proposed for weld seam instance segmentation. First, a Structure-Aware Fusion Module (SAFM) is designed to enhance structural feature representation through strip pooling attention and element-wise multiplicative fusion, targeting the difficulty in extracting elongated and complex features. Second, a C2f-based Boundary-Enhanced Aggregation Module (C2f-BEAM) is constructed to improve edge feature sensitivity by integrating multi-scale boundary detail extraction, feature aggregation, and attention mechanisms. Finally, the inner minimum point distance-based intersection over union (Inner-MPDIoU) is introduced to improve localization accuracy for weld seam regions. Experimental results on the self-built weld seam image dataset show that SABE-YOLO outperforms YOLOv8n-Seg by 3 percentage points in the AP(50–95) metric, reaching 46.3%. Meanwhile, it maintains a low computational cost (18.3 GFLOPs) and a small number of parameters (6.6M), while achieving an inference speed of 127 FPS, demonstrating a favorable trade-off between segmentation accuracy and computational efficiency. The proposed method provides an effective solution for high-precision visual perception of complex weld seam structures and demonstrates strong potential for industrial application.

## 1. Introduction

In modern automated manufacturing, welding robots have been widely adopted in industries such as shipbuilding, automotive engineering, and steel construction [1]. However, the morphological complexity and diversity of weld seams impose higher demands on the accuracy of welding path planning and execution. Traditional welding robots typically rely on laser sensors or structured light vision systems [2] to acquire weld seam profiles in real time through active visual sensing. Despite their effectiveness, such methods face several challenges [3] in practical applications. For instance, arc light, fumes, and spatter during welding can severely degrade image quality. Moreover, the high real-time requirements and system complexity make it difficult to generalize across various working conditions and weld seam types.

To address the aforementioned issues, recent research in both academia and industry has increasingly focused on pre-scanning strategies and passive vision approaches [4], which aim to identify the weld seam region prior to the welding process and thereby provide stable prior information for path planning. Compared to traditional laser tracking techniques, this strategy not only effectively avoids arc interference and visual noise during welding but also significantly reduces the reliance of the system on real-time processing. This provides a controllable foundation for achieving more flexible and robust welding path planning. Against this backdrop, how to accurately extract weld seam regions from pre-scanned images has become a key challenge for improving both welding quality and operational efficiency.

In recent years, deep learning techniques have achieved remarkable breakthroughs in instance segmentation tasks, offering new solutions for the high-precision extraction of weld seam regions. Early deep learning approaches typically adopted two-stage architectures, such as a Mask Region-based Convolutional Neural Network (Mask R-CNN) [5], which combine object detection and mask generation, and demonstrated strong performance on natural images. Subsequently, one-stage methods such as You Only Look At Coefficients (YOLACT) [6] and Segmenting Objects by Locations (SOLO) [7,8] were proposed, significantly improving inference speed while maintaining acceptable accuracy, making them more suitable for industrial scenarios with real-time requirements. More recently, the introduction of Transformer architectures [9] has further enhanced the ability of models to capture long-range dependencies and global contextual information. Transformer-based methods, represented by Mask DETR with Improved Denoising Anchor Boxes (Mask DINO) [10] and Masked-attention Mask Transformer (Mask2Former) [11], have leveraged query-based mechanisms to improve instance representation and mask consistency, achieving state-of-the-art performance across various natural image segmentation tasks.

However, most existing instance segmentation models are designed based on natural image datasets and still exhibit limited generalization capability in complex industrial environments. Weld seam regions typically present elongated structures, complex shapes, and blurred boundaries, which introduce significant performance bottlenecks for conventional segmentation methods. In addition, although Transformer-based approaches offer strong modeling capabilities, they often suffer from a high computational cost and unstable training when handling densely textured areas or small weld seam targets.

Although the generalization ability of existing instance segmentation models in complex industrial scenarios remains limited, YOLOv8-seg [12], as a representative one-stage instance segmentation model, has demonstrated a favorable balance between detection speed and segmentation accuracy on natural image tasks, showing strong potential for engineering applications. This model is optimized based on the design philosophy of YOLACT [6], in which object detection and instance segmentation branches are executed in parallel. Prototype masks and corresponding coefficients are learned and linearly combined to generate efficient mask predictions, providing a solid foundation for further task-specific adaptations in industrial environments.

Considering the limited computational resources of edge welding devices, this study builds a Structure-Aware and Boundary-Enhanced YOLO (SABE-YOLO) network based on the lightweight YOLOv8n-seg, tailored to the practical requirements of weld seam recognition in industrial environments. To address the challenge of extracting features from elongated and structurally complex weld seams, strip pooling [13], attention mechanisms, and element-wise multiplicative fusion [14] are utilized to enhance structural feature perception. Furthermore, to mitigate the issue of blurred weld boundaries, a multi-scale boundary enhancement strategy is designed and integrated with Adaptive Spatial Feature Fusion (ASFF) [15] and an Efficient Multi-scale Attention (EMA) module [16], effectively strengthening sensitivity to boundary details. Finally, the inner minimum point distance-based intersection over union (Inner-MPDIoU) [17,18] loss function is employed to improve segmentation accuracy and robustness under complex weld seam conditions.

Compared to existing mainstream approaches, SABE-YOLO integrates both structure-aware fusion and boundary modeling mechanisms, effectively addressing the limitations of YOLOv8n-seg in capturing the geometric structure of weld seams. Meanwhile, its lightweight and efficient design significantly reduces the modeling redundancy and instability commonly observed in Transformer-based models such as Mask DINO and Mask2Former, which often struggle with weak edge features and small-scale targets in welding scenarios. Overall, SABE-YOLO achieves a better balance among accuracy, robustness, and computational efficiency.

The key contributions of this study can be summarized as follows:

(1) To address the challenges of elongated geometry, complex shapes, and difficult feature extraction in weld seams, a Structure-Aware Fusion Module (SAFM) is proposed. By integrating local directional information with global structural semantics, the module effectively enhances the capacity to capture weld seam geometry.
(2) To mitigate the issue of blurred weld boundaries, a C2f-based Boundary-Enhanced Aggregation Module (C2f-BEAM) is introduced. This module strengthens boundary representation by extracting multi-scale boundary details, thereby improving the perception and recognition of complex boundary structures.
(3) To improve localization accuracy under irregular weld seam shapes, the Inner-MPDIoU loss function is adopted. By constraining the internal overlap between predicted and ground truth boxes based on the minimum point distance, this loss enhances segmentation performance in geometrically complex regions.

## 2. Related Work

### 2.1. Progress of Instance Segmentation in Weld Seam Inspection

Traditional weld seam detection methods primarily rely on image preprocessing, edge detection, and handcrafted feature extraction algorithms, such as the Canny operator [19], Hough transform [20], and grayscale histogram analysis. These classical computer vision approaches played a role in early-stage weld seam recognition but often suffer from poor robustness and limited generalization when applied to industrial weld images characterized by strong reflections, complex backgrounds, and irregular shapes. With the introduction of convolutional neural networks (CNNs), weld seam detection has gradually shifted toward an end-to-end deep learning paradigm, enabling the direct extraction of multi-scale and semantically rich features from raw images, thereby significantly improving recognition performance.

CNN-based object detection models, such as Faster R-CNN [21], the YOLO series [12,22,23,24], and RetinaNet [25], have been widely applied to weld seam localization tasks, achieving a favorable balance between detection accuracy and real-time performance. However, weld seams typically exhibit elongated and variable geometries, blurred boundaries, and susceptibility to interference. As a result, traditional bounding box-based detection methods often fall short of meeting the pixel-level localization requirements of practical welding systems. To address these limitations, research has gradually shifted toward instance segmentation approaches, including Mask R-CNN [5], SOLOv2 [8], YOLACT [6], and Mask2Former [11]. These methods not only predict the location of weld seams but also generate precise masks for each instance, providing a solid theoretical foundation for high-precision recognition in complex weld seam regions.

In recent studies, Gao et al. [26] proposed an improved version of YOLOv5 by integrating re-parameterized convolutional networks and attention mechanisms, enabling more accurate localization of weld feature points under high-noise conditions. Zou et al. [27] designed a lightweight SOLOv2-based architecture for weld seam instance segmentation, effectively improving both inference speed and segmentation quality. Zhao et al. [28] combined YOLOv8s-seg with a MobileNetV3 backbone to achieve real-time instance mask segmentation of weld seams on resource-constrained devices. These methods have been validated in complex industrial welding environments. Although challenges such as background interference and weld shape variability remain, structural customization and transfer learning strategies have significantly enhanced model adaptability and robustness in task-specific welding scenarios.

### 2.2. Application of Attention Mechanisms in Instance Segmentation

The introduction of attention mechanisms into instance segmentation tasks has significantly improved the accuracy of object localization and boundary recognition. Compared to traditional CNN architectures, attention modules guide the network to focus on more discriminative region features while suppressing redundant background information, thereby enabling the extraction of more representative mask representations in complex images. Among these, spatial attention and channel attention mechanisms have been widely incorporated into classical segmentation architectures. For instance, Woo et al. [29] proposed the Convolutional Block Attention Module (CBAM), which combines spatial and channel attention to perform importance weighting along feature map dimensions, and has been extensively adopted to enhance the segmentation performance of models such as Mask R-CNN [5]. Hu et al. [30] introduced the Squeeze-and-Excitation Network (SE-Net), which dynamically recalibrates feature channels through channel attention and significantly enhances the response to salient object regions. These mechanisms have not only shown strong performance in natural image segmentation but also demonstrated good generalization in industrial instance segmentation scenarios.

As the core of Transformer architectures, the self-attention mechanism [9] has shown great potential in instance segmentation tasks in recent years. Representative methods such as SegFormer [31] and Mask2Former [11] incorporate self-attention modules to model long-range dependencies and global contextual information, effectively enhancing the ability of the network to capture structural relationships between objects. In particular, Mask2Former introduces a unified mask prediction framework that combines self-attention mechanisms with point-based prediction strategies, achieving performance significantly superior to traditional CNN-based models on benchmark datasets such as COCO and ADE20K.

In summary, attention mechanisms provide more robust and accurate modeling capabilities for addressing challenges such as complex backgrounds, multi-object occlusions, and blurred boundaries in instance segmentation tasks. They also offer essential technical support for the generation and refinement of instance masks in complex industrial imaging scenarios.

## 3. Materials and Methods

### 3.1. Dataset

#### 3.1.1. Data Acquisition and Analysis

The acquisition and analysis of weld seam images not only provide high-quality data support for deep learning models but also facilitate the investigation of how various weld geometries, defect features, and environmental factors affect detection performance. This forms a foundation for the optimization and practical deployment of intelligent weld inspection systems. In this study, the weld seam image dataset was collected from a heavy machinery manufacturing company located in Guilin, Guangxi, China.

**Figure 1:**
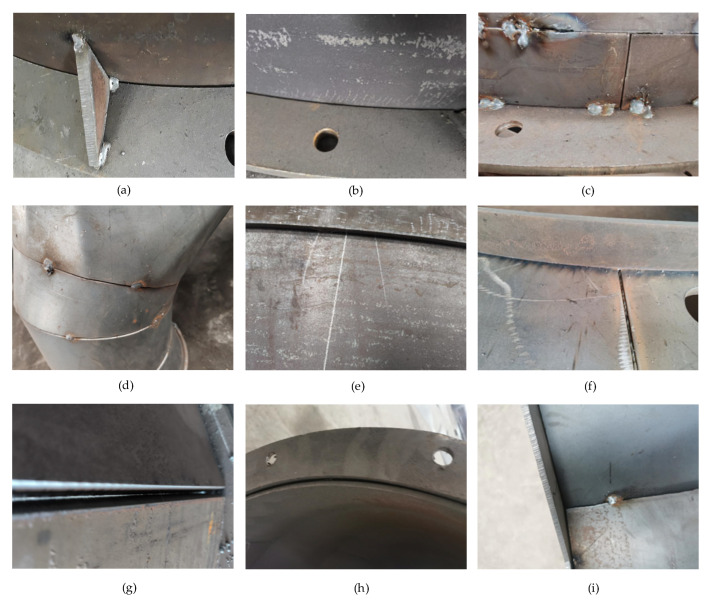
Representative samples from the weld seam dataset. (**a**) Typical fillet weld with vertical plate support; (**b**) flat-position fillet weld; (**c**) mixed joint with butt and fillet welding; (**d**) pipe-to-pipe butt weld; (**e**) narrow-gap linear butt weld; (**f**) corner joint with partial fillet and butt weld; (**g**) wide-gap butt weld; (**h**) circular butt weld on cylindrical structure; and (**i**) edge-position fillet weld near corner.

The dataset used in this study contains various types of weld seams on complex workpieces, including butt welds, fillet welds, plug welds, and seam welds. All images were captured at a resolution of 4064 × 3048 pixels and saved in jpg format. To ensure diversity and better simulate real-world welding conditions, the dataset includes weld images captured under varying distances, angles, lighting conditions, and levels of occlusion. Each image contains multiple types of weld seams to increase scene complexity. A combination of different shooting angles, distances, illumination intensities, and occlusions was employed during image acquisition. A total of 1978 images were collected. The dataset was divided into training, validation, and test sets at a ratio of 6:2:2, resulting in 1186 images for training, 396 for validation, and 396 for testing. Representative examples from the dataset are shown in Figure 1.

In instance segmentation tasks, insufficient training data often leads to overfitting issues [32]. Moreover, the collected weld seam images may not comprehensively represent the diverse conditions encountered in welding environments, such as varying illumination levels, noise interference, image sharpness, and occlusions. To ensure robust model performance during both training and testing, multiple data augmentation techniques [33] were applied to the training and validation sets. These techniques included random Gaussian noise addition, brightness adjustment, cropping, translation, rotation, flipping, and Cutout. By simulating complex data distributions and noise patterns typical of welding environments, these augmentations enhanced the generalization capability and practical applicability of the model. After augmentation, the training set was expanded to 7113 images, while the validation and test sets contained 396 images each.

#### 3.1.2. Generalization Validation Dataset: Crack Segmentation Dataset

Currently, there is no publicly available benchmark dataset specifically for weld seam instance segmentation, which to some extent limits the validation and comparison of relevant models across different scenarios. To evaluate the generalization capability of the proposed SABE-YOLO model in terms of structure awareness and boundary modeling, this study introduces the Crack Segmentation Dataset [34] as an external validation platform.

The Crack Segmentation Dataset, released by Roboflow Universe, consists of 4029 static images covering various crack scenarios on road and wall surfaces, and provides precise pixel-level instance mask annotations. It is widely used in traffic infrastructure monitoring and autonomous driving vision systems. The dataset is divided into a training set (3717 images), a validation set (200 images), and a test set (112 images), each accompanied by accurate pixel-level mask annotations.

**Figure 2:**
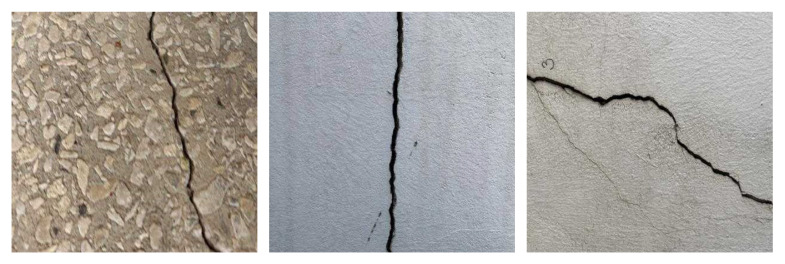
Representative samples from the Crack Segmentation Dataset.

Although cracks and weld seams differ in semantic categories, they share common visual characteristics such as elongated structures, blurred boundaries, and irregular shapes, making them structurally analogous in the context of structure-aware networks. Representative examples from the dataset are shown in Figure 2.

Accordingly, 100 crack images with structural similarities to weld seams were selected from the validation and test sets of the Crack Segmentation Dataset. Under zero-shot inference conditions, the generalization performance of the SABE-YOLO model was evaluated, focusing on its boundary recognition and structural retention capabilities in non-welding scenarios, and quantitatively compared with mainstream segmentation models.

### 3.2. Methods

#### 3.2.1. SABE-YOLO Network Architecture

**Figure 3:**
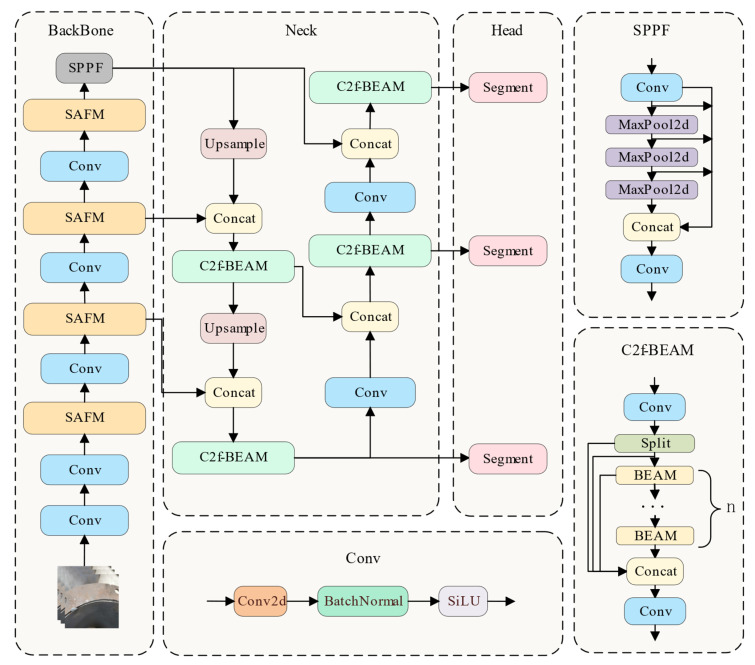
Overview of the SABE-YOLO Network.

As shown in Figure 3, the SABE-YOLO model is built upon YOLOv8n-seg and consists of three main components: the Backbone, Neck, and Head. Structure-aware and boundary-enhanced mechanisms are integrated into each stage to improve the capability of the model in weld seam instance segmentation tasks.

In the Backbone stage, the model performs layer-by-layer feature extraction on the input weld seam image to generate multi-scale semantic feature maps. To enhance perception of elongated weld structures, four SAFMs are integrated into the backbone and embedded after multiple convolutional blocks. SAFMs utilize direction-aware strip pooling and a dual-branch fusion mechanism to strengthen the ability of the model to capture geometric structures of welds while maintaining low computational overhead.

During the Neck stage, feature maps from different scales are fused and transmitted. The original C2f modules in YOLOv8 are replaced by three C2f-BEAMs, which progressively enhance boundary feature representation. On top of channel-wise feature aggregation, these modules incorporate BEAM to extract blurry boundary information of weld seams. Additionally, ASFF and EMA mechanisms are integrated to further improve responsiveness to key edge regions.

In the Head stage, the fused features are used to simultaneously predict the position, class, and segmentation mask of weld targets. To improve the accuracy of fitting irregular weld shapes, an Inner-MPDIoU loss function is introduced in the bounding box regression branch, replacing the original CIoU loss. This loss applies minimal distance constraints between inner points of the predicted and ground truth boxes to ensure internal geometric consistency, thereby enhancing localization robustness, particularly for complex-shaped and boundary-blurred weld seams.

**Figure 4:**
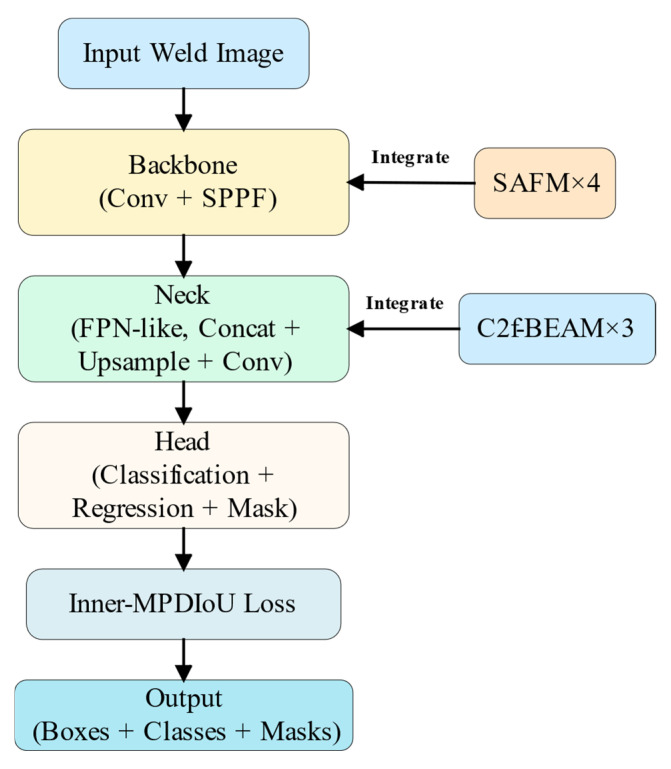
High-level schematic of SABE-YOLO illustrating the integration positions of SAFM, C2f-BEAM, and Inner-MPDIoU into the YOLOv8n-seg framework.

Through modular integration of these three improvements within the YOLO framework, SABE-YOLO significantly enhances structural perception, boundary delineation, and object localization accuracy, all while maintaining a lightweight inference cost. A high-level architectural diagram of SABE-YOLO is shown in Figure 4. In the following sections, we provide detailed descriptions of the three key modules: SAFM, C2f-BEAM, and the Inner-MPDIoU loss function.

#### 3.2.2. Structure-Aware Fusion Module (SAFM)

**Figure 5:**
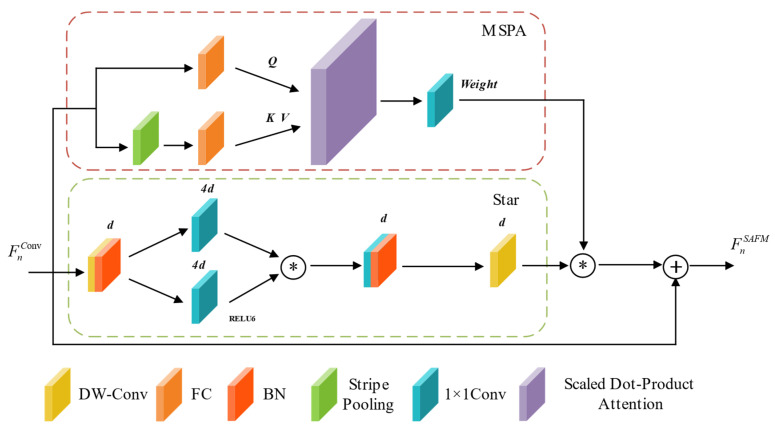
Network structure of SAFM. The red dashed box indicates the MSPA branch, where *Weight* denotes the output of the branch. The green dashed box represents the Star branch. *d* indicates the input feature dimension, and ReLU6 is used as the activation function. “*” denotes element-wise multiplication; “+” denotes element-wise addition. The input feature $F_{n}^{Conv}\in R^{w_{n}\times h_{n}\times c_{n}}$ is extracted, where $w_{n}$, $h_{n}$, and $c_{n}$ represent the spatial dimensions and channel number at stage *n* in SAFM (*n* = 1, 2, 3, 4). The corresponding output feature $F_{n}^{SAFM}$ represents the enhanced feature extracted by SAFM.

The SAFM is proposed to enhance the feature extraction capability of the weld segmentation network and to improve the localization of weld seams. Its position within the overall network is shown in Figure 3, and its detailed structure is illustrated in Figure 5.

Considering that most weld seams exhibit elongated and fine structures, a Multi-head Strip Pooling Attention (MSPA) branch is introduced in SAFM. Strip pooling [13] and self-attention computation [9] are incorporated in this branch to capture fine-grained spatial structures and to enhance the response to boundary regions of slender targets. The computation of MSPA is defined in Equations (1) and (2) [9].

(1) $$\mathrm{MSPA}(Q,K,V)=\mathrm{Reweight}\left(\mathrm{Concat}\left({\mathrm{head}}_{0},\ldots ,{\mathrm{head}}_{N}\right)W^{O}\right)$$

(2) $${\mathrm{head}}_{i}=\mathrm{Attention}\left(QW_{i}^{Q},SP(K)W_{i}^{K},SP(V)W_{i}^{V}\right)$$

Here, $W^{O}\in R^{C\times C}$, $W_{i}^{Q}\in R^{C\times d_{head}}$, $W_{i}^{K}\in R^{C\times d_{head}}$ and $W_{i}^{V}\in R^{C\times d_{head}}$ represent the linear projection weight matrices. *N* denotes the number of attention heads. Concat refers to the concatenation of features along the channel dimension, while *Reweight* indicates the use of 1 × 1 convolution to compress projected features into weighting vectors. *SP* stands for strip pooling, whose formulation is presented in Equation (3) [13].

(3) $$SP(x)=\mathrm{Norm}\left(\mathrm{Concat}\left({\mathrm{reshape}}_{h}(x)W^{S},{\mathrm{reshape}}_{v}(x)W^{S}\right)\right)$$

Let the input feature be denoted as $x\in R^{H\times W\times C}$. Horizontal strip pooling yields $x_{h}=reshape_{h}(x)$, where $x_{h}\in R^{W\times 1\times C}$; vertical pooling yields $x_{v}=reshape_{v}(x)$, where $x_{v}\in R^{H\times 1\times C}$. The parameter $W^{S}$ represents a linear transformation, and Concat refers to concatenation along the channel dimension. The attention computation is defined in Equation (4) [9].

(4) $$\mathrm{Attention}(Q,K,V)=\mathrm{Softmax}\left(\frac{QK^{T}}{\sqrt{d_{\mathrm{head}}}}\right)V$$

In addition, to enhance the receptive field of the network, a Star branch inspired by StarBlock [14] is introduced into SAFM. By employing multi-scale feature expansion and element-wise interaction, the receptive field and feature representation capacity of the model are improved, thereby enhancing its ability to perform semantic modeling in complex structural regions. Specifically, the Star branch utilizes element-wise multiplication to fuse subspace features, instead of increasing the network width (i.e., number of channels) as in conventional neural networks, enabling high-dimensional representations to be generated within a low-dimensional computational space.

As illustrated in Figure 5, in the Star branch, the input feature map is first processed by a depthwise convolution to extract local features and reduce computational complexity. The resulting features are then split into two parallel paths, each undergoing a 1 × 1 convolution to adjust the number of channels in preparation for element-wise multiplication. One path employs the ReLU6 [35] activation function to enhance non-linear expressiveness, while the other remains a linear transformation to retain more original information. Subsequently, the feature maps from both paths are fused via element-wise multiplication, enabling subspace feature interaction and facilitating the extraction of higher-dimensional weld representations.

After the element-wise multiplication, a 1 × 1 convolution is applied to restore the number of channels, followed by a depthwise convolution to further refine local features. Finally, the output of the Star branch is fused multiplicatively with the MSPA features and then added to the residual connection to jointly capture both high-dimensional implicit features and the elongated structural characteristics of the weld seam.

#### 3.2.3. C2f-Based Boundary-Enhanced Aggregation Module (C2f-BEAM)

In the segmentation of weld seam targets, multi-scale boundary detail information plays a critical role, especially under conditions of blurred boundaries and complex shapes. To address this challenge, inspired by the Multi-scale Detail-enhanced Segment Anything Model (MDSAM) [36], a Boundary-Enhanced Aggregation Module (BEAM) is proposed.

BEAMs highlight weld seam boundary details across multiple scales, enabling the model to capture global contours while focusing on local features, thereby enhancing its perception of weld targets at various scales. By explicitly reinforcing attention to boundary regions, BEAMs effectively reduce the blurred transition between weld seams and the background, improving segmentation accuracy. Furthermore, through the aggregation of multi-scale boundary features, the module enhances adaptability to complex weld geometries and strengthens boundary recognition capability.

**Figure 6:**
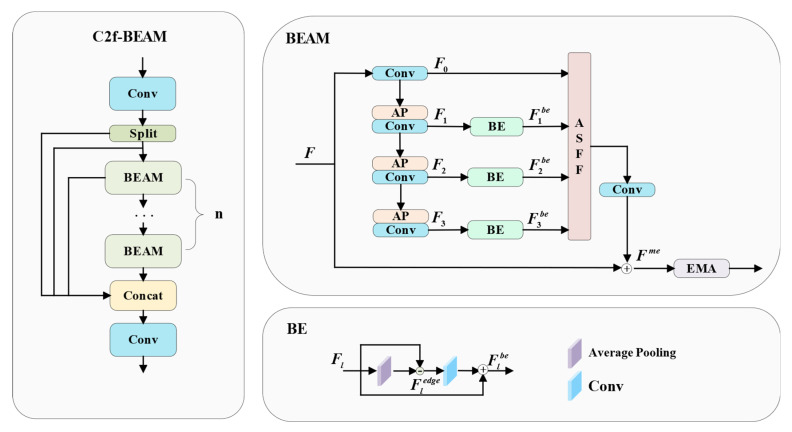
Network architecture of C2f-BEAM. The BEAM is a submodule of C2f-BEAM, and BE represents a subcomponent of the BEAM.

To better integrate multi-scale boundary detail information, the ASFF module is introduced for multi-scale feature aggregation, combined with the EMA mechanism to enhance the representation of critical detail features. Based on this, the BEAM is embedded into the C2f block of the neck network, resulting in the proposed C2f-BEAM with enhanced boundary representation and feature aggregation capabilities. The structure of the C2f-BEAM used in this study is illustrated in Figure 6.

As illustrated in Figure 6, C2f-BEAM processes the input data through two branches. One branch is directly passed to the output, while the other is processed by multiple BEAMs. This dual-branch design enhances the network’s nonlinearity and representational capacity. Within each BEAM, the input feature map $F\in R^{C\times H\times W}$ is first downsampled using a 3 × 3 average pooling followed by a 1 × 1 convolution to extract multi-scale detail features. Then, a Boundary Enhancement (BE) module is employed to highlight weld seam boundary information. Subsequently, multi-scale features are adaptively fused and combined with a residual connection. The multi-scale boundary enhancement process is formulated in Equations (5)–(9) [36].

(5) $$F_{0}=\phi_{1\times 1}(F)$$

(6) $$F_{t+1}=AP\left(\phi_{1\times 1}\left(F_{t}\right)\right),(0\leq t\leq 2)$$

(7) $$F_{l}^{edge}=F_{l}-AP\left(F_{l}\right)$$

(8) $$F_{l}^{be}=\phi_{1\times 1}^{\prime }(F_{l}^{edge})+F_{l}$$

(9) $$F^{me}=F+\phi_{1\times 1}(ASFF(F_{0},F_{1}^{be},F_{2}^{be},F_{3}^{be}))$$

Let $F_{t}\in R^{C\times H\times W}$ denote the feature map at scale *t* ∈ {0, 1, 2, 3}, and let $\phi_{1\times 1}$ denote the convolution layer with a 1 × 1 kernel. The average pooling module *AP* consists of a convolutional layer with a 3 × 3 kernel and performs average pooling. $F_{l}$ represents the hierarchical input feature from the BE module, while $F^{edge}$ denotes the edge feature map. The adaptive spatial feature fusion *ASFF* [15] integrates multi-scale features. The resulting feature map $F^{me}$ is obtained by combining boundary enhancement and multi-scale feature aggregation. The fusion process is formulated in Equations (10)–(14) [15].

(10) $$F^{ASFF}=\alpha F_{0}+\beta F_{1}+\gamma F_{2}+\mu F_{3}$$

(11) $$\alpha =\frac{e^{\lambda_{\alpha }}}{e^{\lambda_{\alpha }}+e^{\lambda_{\beta }}+e^{\lambda_{\gamma }}+e^{\lambda_{\mu }}}$$

(12) $$\beta =\frac{e^{\lambda_{\beta }}}{e^{\lambda_{\alpha }}+e^{\lambda_{\beta }}+e^{\lambda_{\gamma }}+e^{\lambda_{\mu }}}$$

(13) $$\gamma =\frac{e^{\lambda_{\gamma }}}{e^{\lambda_{\alpha }}+e^{\lambda_{\beta }}+e^{\lambda_{\gamma }}+e^{\lambda_{\mu }}}$$

(14) $$\mu =\frac{e^{\lambda_{\mu }}}{e^{\lambda_{\alpha }}+e^{\lambda_{\beta }}+e^{\lambda_{\gamma }}+e^{\lambda_{\mu }}}$$

Let $F_{t}\in R^{C\times H\times W}$ denote the input feature map at scale *t*, where *t* ∈ {0, 1, 2, 3}. The weight coefficients *α*, *β*, *γ* and *μ* are obtained by applying a 1 × 1 convolution to the input features at each scale. These coefficients are then concatenated along the channel dimension and normalized via a softmax operation, ensuring that their values lie in the range [0, 1] and sum to 1.

Finally, the boundary detail features of weld seams are further enhanced through the EMA module [16], which is illustrated in the following figure.

**Figure 7:**
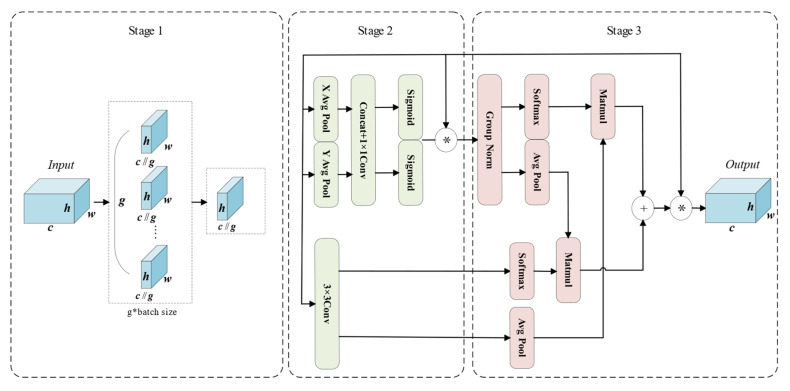
Architecture of the EMA module. The first stage performs feature grouping, the second stage consists of parallel subnetworks, and the third stage conducts cross-spatial learning. “*” denotes element-wise multiplication; “+” denotes element-wise addition.

As illustrated in Figure 7, the EMA module consists of three main stages. In the first stage, feature grouping is performed on the weld seam features. For any given input feature map $X\in R^{C\times H\times W}$, EMA divides it into *G* sub-feature groups, each learning semantic representations independently. This grouping strategy enables more efficient allocation of GPU resources and facilitates parallel processing. Moreover, it enhances semantic-aware feature learning while suppressing noise. The grouping process is formulated in Equation (15) [16].

(15) $$X=[X_{0},X_{1},X_{2},\ldots ,X_{G}],X\in R^{C\times H\times W}$$

In the second stage, parallel sub-networks are employed, where EMA extracts attention-based weighting descriptors from grouped feature maps via three parallel primary paths and one residual connection path. The first two main paths first apply 1 × 1 convolutions to encode channel-wise information along the *X* and *Y* directions, followed by one-dimensional global average pooling. The pooled feature maps are then concatenated and passed through another 1 × 1 convolution. Subsequently, the features are split again along the *X* and *Y* axes to capture directional channel dependencies and activated using the Sigmoid function to generate two channel-wise attention maps. These attention maps are then element-wise multiplied with the grouped input features from the residual connection path, enhancing the channel representations and producing enriched weld seam features. In the third path, a single 3 × 3 convolution is applied to capture multi-scale weld seam features, which are utilized in the subsequent cross-spatial learning stage. At this stage, EMA not only encodes inter-channel dependencies to adaptively recalibrate channel importance but also effectively preserves precise spatial structure information within each channel, thereby enhancing sensitivity to spatial details.

The third stage is the cross-spatial learning stage. First, the multi-scale weld seam feature maps output from the 3 × 3 branch in the second stage are encoded with global spatial information via global average pooling. These pooled features are then aggregated with the outputs of the two 1 × 1 branches, which have been normalized using group normalization, through matrix dot-product operations to generate the first spatial attention map. Simultaneously, the outputs of the two 1 × 1 branches are subjected to two-dimensional global average pooling to encode their respective global spatial representations, while the output of the 3 × 3 branch is directly reshaped to the corresponding dimension. These representations are further fused via matrix dot-product operations to produce the second spatial attention map.

Finally, the output feature maps within each group are aggregated using the two spatial attention weights generated by the Sigmoid function, thereby further enhancing the representation of weld seam boundary information.

#### 3.2.4. Inner Minimum Point Distance Based Intersection over Union (Inner-MPDIoU)

In welding scenarios, one of the key tasks in weld seam instance segmentation is to accurately determine the boundary frame of the weld seam in the image. To improve segmentation accuracy and accelerate model convergence, the loss function used for training was refined. In the original YOLOv8n-seg, the Complete Intersection over Union (CIoU) [37] was employed as the coordinate loss function. CIoU is an extension of the Distance Intersection over Union (DIoU) [38] loss, incorporating the aspect ratio of the predicted bounding box to further enhance localization accuracy. The computation of CIoU is formulated in Equations (16)–(18) [37].

(16) $$Loss_{CIoU}=1-IoU+\frac{\rho^{2}(b,b^{gt})}{c^{2}}+\alpha \nu$$

(17) $$\nu =\frac{4}{\pi^{2}}{\left(\arctan\frac{w^{gt}}{h^{gt}}-\arctan\frac{w}{h}\right)}^{2}$$

(18) $$\alpha =\frac{\nu }{(1-IoU)+\nu }$$

**Figure 8:**
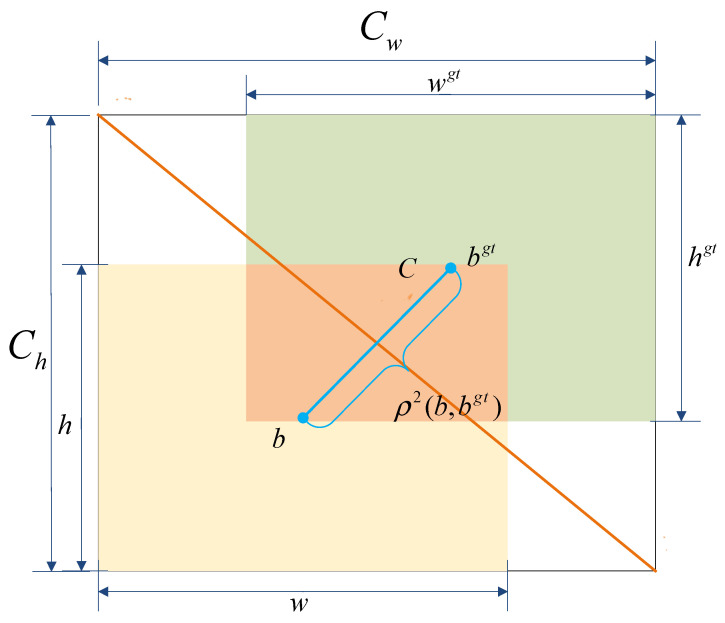
Schematic illustration of the CIoU computation structure.

The term IoU denotes the intersection over union between the predicted box and the ground truth box. The symbol *ρ* represents the Euclidean distance between the centers of the two boxes. The parameters b and $b^{gt}$ denote the centers of the predicted and ground truth boxes, respectively, while w and h are the width and height of the predicted box and $w^{gt}$ and $h^{gt}$ correspond to those of the ground truth box. c denotes the diagonal length of the smallest enclosing box that contains both the predicted and ground truth boxes. The coefficient *α* serves as a balancing factor for the weighted terms, and *v* evaluates the consistency of the aspect ratios between the predicted and ground truth boxes. The structure is illustrated in Figure 8.

However, although CIoU adjusts the weight of the center distance term dynamically, the computation of *v* heavily depends on the aspect ratio difference. This leads to reduced sensitivity in detecting changes in extreme aspect ratios. For example, in the case of slender weld seam targets, the contribution of the center distance term may be excessively amplified. As a result, the optimization direction can be negatively affected. In addition, CIoU relies on aspect ratio similarity to measure shape differences. However, in complex welding scenarios, the shape of weld seams varies significantly. Relying solely on aspect ratio is not sufficient for accurate shape alignment.

To address the limitations of CIoU, a novel loss function named Inner-MPDIoU is proposed, drawing inspiration from both Inner-IoU and MPDIoU. Inner-MPDIoU introduces a scaling factor to reduce the sizes of the predicted and ground truth boxes, thereby focusing on their internal regions and mitigating the interference of aspect ratio in center-based weight distribution. Moreover, it calculates the minimum point-to-point distance between the two boxes directly, eliminating the reliance on aspect ratio and ensuring a more stable and consistent weighting scheme. The computational structure of Inner-MPDIoU is illustrated in Figure 8.

**Figure 9:**
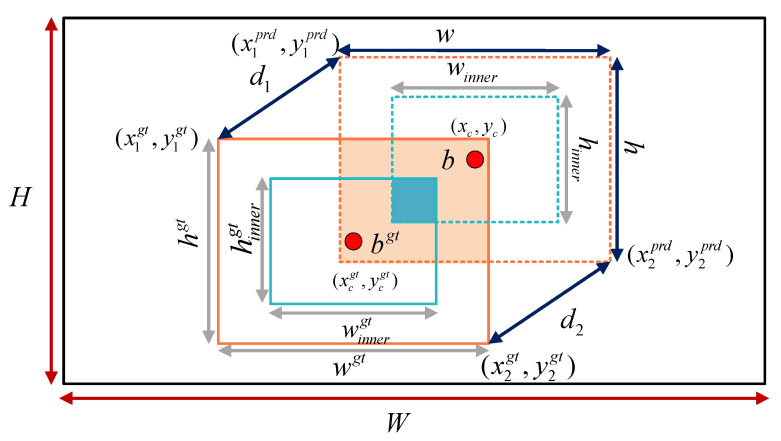
Schematic diagram of the Inner-MPDIoU computation structure.

As shown in Figure 9, *w* and *h* represent the width and height of the predicted bounding box, while $w^{gt}$ and $h^{gt}$ denote those of the ground truth box. b and $b^{gt}$ refer to the center points of the predicted and ground truth boxes, respectively. The center of the ground truth box is denoted by coordinates $\left(x_{c}^{gt},y_{c}^{gt}\right)$ and the predicted box center by $\left(x_{c},y_{c}\right)$.

The auxiliary bounding box of the predicted box is obtained by scaling its dimensions with a reduction factor, resulting in width $w_{inner}$ and $h_{inner}$. Similarly, the auxiliary box of the ground truth is defined with dimensions $w_{inner}^{gt}$ and $h_{inner}^{gt}$. *W* and *H* represent the width and height of the input feature map.

The two opposite corner points of the predicted box are denoted as $\left(x_{1}^{prd},y_{1}^{prd}\right)$ and $\left(x_{2}^{prd},y_{2}^{prd}\right)$, with constraints $x_{2}^{prd}>x_{1}^{prd}$ and $y_{2}^{prd}>y_{1}^{prd}$. Likewise, the two opposite corner points of the ground truth box are denoted as $\left(x_{1}^{gt},y_{1}^{gt}\right)$ and $\left(x_{2}^{gt},y_{2}^{gt}\right)$, satisfying $x_{2}^{gt}>x_{1}^{gt}$ and $y_{2}^{gt}>y_{1}^{gt}$.

The shortest distance between points $\left(x_{1}^{prd},y_{1}^{prd}\right)$ and $\left(x_{1}^{gt},y_{1}^{gt}\right)$ is denoted as $d_{1}$, while the shortest distance between points $\left(x_{2}^{prd},y_{2}^{prd}\right)$ and $\left(x_{2}^{gt},y_{2}^{gt}\right)$ is denoted as $d_{2}$. The loss function based on Inner-MPDIoU is formulated as follows:

(19) $$b_{l}^{gt}=x_{c}^{gt}-\frac{w^{gt}*ratio}{2},b_{r}^{gt}=x_{c}^{gt}+\frac{w^{gt}*ratio}{2}$$

(20) $$b_{t}^{gt}=y_{c}^{gt}-\frac{h^{gt}*ratio}{2},b_{b}^{gt}=y_{c}^{gt}+\frac{h^{gt}*ratio}{2}$$

(21) $$b_{l}=x_{c}-\frac{w*ratio}{2},b_{r}=x_{c}+\frac{w*ratio}{2}$$

(22) $$b_{t}=y_{c}-\frac{h*ratio}{2},b_{b}=y_{c}+\frac{h*ratio}{2}$$

(23) $$inter=(\min(b_{r}^{gt},b_{r})-\max(b_{l}^{gt},b_{l}))*(\min(b_{b}^{gt},b_{b})-\max(b_{t}^{gt},b_{t}))$$

(24) $$union=(w^{gt}*h^{gt})*{\left(ratio\right)}^{2}+(w*h)*{\left(ratio\right)}^{2}-inter$$

(25) $$IoU^{inner}=\frac{inter}{union}$$

(26) $$d_{1}^{2}={\left(x_{1}^{prd}-x_{1}^{gt}\right)}^{2}+{\left(y_{1}^{prd}-y_{1}^{gt}\right)}^{2}$$

(27) $$d_{2}^{2}={\left(x_{2}^{prd}-x_{2}^{gt}\right)}^{2}+{\left(y_{2}^{prd}-y_{2}^{gt}\right)}^{2}$$

(28) $$InnerMPDIoU=IoU^{inner}-\frac{d_{1}^{2}}{H^{2}+W^{2}}-\frac{d_{2}^{2}}{H^{2}+W^{2}}$$

(29) $$Loss_{InnerMPDIoU}=1-InnerMPDIoU$$

In Equations (19)–(25) [17], *ratio* denotes the scaling factor, typically ranging from [0.5, 1.5]. The parameters $b_{l}$, $b_{r}$, $b_{t}$, and $b_{b}$ represent the coordinates of the left, right, top, and bottom edges of the predicted box after applying the scaling factor, following the same procedure as for the ground truth box.

Unlike the conventional IoU computation, Inner-IoU first uses the scaling factor *ratio* to resize the predicted and ground truth boxes to generate two auxiliary boxes. Then, it computes the intersection over union of these auxiliary boxes, denoted as $IoU^{inner}$.

In SABE-YOLO, the value of *ratio* is set to 0.9, which helps the model focus on the core internal region of the weld seam, reducing background noise interference and enabling pixel-level localization of narrow weld seams.

In Equations (26)–(29) [18], the squared distances between the top-left and bottom-right corners of the predicted and ground truth bounding boxes are first computed as $d_{1}^{2}$ and $d_{2}^{2}$, respectively. Then, the penalty terms $\frac{d_{1}^{2}}{H^{2}+W^{2}}$ and $\frac{d_{2}^{2}}{H^{2}+W^{2}}$ are introduced into the loss function.

Here, the normalization term $H^{2}+W^{2}$ represents the sum of the squared height and width of the input image and is used to eliminate the influence of image scale on the corner point distances, thereby enhancing the consistency and stability of the loss.

Typically, a rectangular bounding box can be uniquely determined by the coordinates of its top-left and bottom-right corner points. Traditional bounding box regression methods improve localization accuracy by optimizing metrics such as non-overlapping area, center point distance, and aspect ratio deviation. However, the effectiveness of these metrics relies heavily on the precision of corner point coordinates. To address this limitation, MPDIoU directly constrains the distances between the corner points of predicted and ground truth boxes, thereby enhancing the training performance of bounding box regression. This method not only enables more accurate measurement of spatial deviation between predicted and annotated boxes but also facilitates the implicit learning of weld seam rotation features. As a result, the segmentation mask is guided to adaptively adjust its orientation, ultimately leading to improved robustness in identifying weld seams with varying rotation angles.

In summary, the scaling operation enhances the robustness of the loss function to variations in object scale, while the minimum distance point loss provides a clear gradient direction. The combination of both in Inner-MPDIoU leads to smoother gradient propagation, thereby accelerating model convergence and reducing fluctuations in the final segmentation accuracy.

#### 3.2.5. Weld Seam Instance Segmentation Method Based on SABE-YOLO

Algorithm 1 illustrates the inference process of the weld seam instance segmentation method based on the SABE-YOLO network. Given an input weld image containing various types such as flat welds, fillet welds, and butt welds, multi-scale feature maps are first extracted using the backbone network. SAFM is introduced to balance computational efficiency and enhance the backbone’s perception of slender weld structures.

Subsequently, the neck network incorporates C2f-BEAM to highlight weld boundary details, while ASFF mechanism aggregates features across scales. Combined with EMA module, the representation of key edge information is further strengthened. In the detection head, fixed-structure convolutional layers are used to directly predict class scores, bounding box offsets, and mask coefficients. Specifically, the classification branch outputs the category probabilities of welds, the regression branch decodes grid coordinates to generate bounding boxes, and the mask branch dynamically generates instance segmentation results based on the prototype features.

Finally, Non-Maximum Suppression (NMS) is applied to eliminate redundant boxes, and pixel-level weld contours are reconstructed using a mask assembly algorithm [6] to visualize the segmentation output.
**Algorithm 1: Inference Algorithm of Weld Seam Segmentation Based on SABE-YOLO****Input**: An input weld seam image *I* with shape H × W × 3. **Output**: Predicted class labels, bounding boxes, and instance masks 1: multi_scale_features = extract_SAFM_backbone(I) 2: pyramid_features = PANet_BEAM-fusion(multi_scale_features) 3: **for each** scale_feature in pyramid_features do: 4:         grid_centers = generate_grid_centers(scale_feature) 5:         cls_feat, reg_feat = depthwise_sep_conv(scale_feature) 6:         pred_scores = class_conv(cls_feat) 7:         bbox_preds = bbox_conv(reg_feat) 8:         mask_coeff = mask_conv(reg_feat) 9: **End for** 10: all_preds = concat([pred_scores, bbox_preds, mask_coeff]) 11: decoded_boxes = decode_bbox(grid_centers, bbox_preds) 12: topk_indices = select_topk(all_preds) 13: nms_results = NMS(decoded_boxes, topk_indices) 14: final_masks = assemble_masks(mask_proto, mask_coeff) 15: **return** nms_results.classes, nms_results.boxes, final_masks

## 4. Experiments and Results

### 4.1. Experimental Environment and Parameters

All experiments were conducted using the PyTorch 2.1.2 deep learning framework. The hardware configuration consisted of an AMD Ryzen 9 5900X 12-Core Processor (3.70 GHz) and an NVIDIA GeForce RTX 4080 SUPER GPU. The operating system was Windows 10, with Python 3.8.10, CUDA 12.1, and cuDNN 8.9.6.

During training, the input image size was set to 640 × 640 pixels. A batch size of 16 and 8 data loading workers were used. The initial learning rate was 0.01, with a weight decay of 0.0005 and a momentum factor of 0.937. The Stochastic Gradient Descent (SGD) was adopted as the optimizer. The total number of training epochs was 300, and the mosaic augmentation was disabled during the final 10 epochs.

### 4.2. Evaluation Metrics

To evaluate the performance of weld seam segmentation, the following metrics are employed: Precision (P), Recall (R), F1-score (F1), Average Precision (AP), number of parameters, GFLOPs (Giga Floating Point Operations Per Second), and inference speed measured in Frames Per Second (FPSs). These metrics provide a comprehensive evaluation of the model in terms of accuracy, robustness, and computational efficiency.

(30) $$P=\frac{T_{p}}{T_{p}+F_{p}}\times 100\%$$

(31) $$R=\frac{T_{p}}{T_{p}+F_{N}}\times 100\%$$

(32) $$F_{1}=\frac{2PR}{P+R}\times 100\%$$

(33) $$AP=\int_{0}^{1}P(R)dR$$

In Equations (30)–(33), *TP* denotes the number of correctly predicted positive samples, *FP* represents the number of negative samples incorrectly predicted as positive, and *FN* indicates the number of positive samples incorrectly predicted as negative. The $F_{1}$ is the harmonic mean of precision and recall, which balances the impact of extreme values from either metric and provides a more comprehensive performance measure.

In the YOLO-series models, precision (P) and recall (R) are evaluated under the conditions of an IoU threshold of 0.5 and a confidence threshold of 0.25, representing the accuracy and completeness of instance segmentation, respectively. The AP (Average Precision) is defined as the area under the precision–recall (P–R) curve, while AP(50–95) refers to the mean of AP values calculated at IoU thresholds ranging from 0.50 to 0.95. This metric comprehensively reflects performance across varying levels of detection difficulty and serves as a key indicator for evaluating overall segmentation accuracy in weld seam instance segmentation tasks.

### 4.3. Performance Comparison Between SABE-YOLO and Mainstream Instance Segmentation Models

Through a series of improvements based on YOLOv8n-seg, the segmentation accuracy was significantly enhanced. To further validate the effectiveness of SABE-YOLO, a comparative experiment was conducted against other mainstream instance segmentation models on the same dataset. Evaluation metrics included Precision (P), Recall (R), Average Precision (AP(50–95)), the number of parameters, Giga Floating-Point Operations per Second (GFLOPs), and inference speed measured in Frames Per Second (FPSs). The results are summarized in Table 1.

To ensure a comprehensive comparison, we additionally include the Improved YOLOv8s-seg model proposed in [12], which was specifically designed for weld seam instance segmentation. As the source code for this method has not been released, we reimplemented the model based on the network architecture and key parameters described in the original paper. Although every effort was made to faithfully reproduce the original design, the absence of certain training details and data augmentation strategies may result in performance deviations from the reported results. Therefore, the experimental outcomes of this model are presented for reference purposes only.

As shown in Table 1, SABE-YOLO achieved the highest AP(50–95) score of 46.3%, outperforming Improved YOLOv8s-seg [28], YOLACT, YOLOv8n-seg, YOLOv11n-seg [23], YOLOv12n-seg [24], Mask-DINO, Mask Transfiner [39], Mask2Former, and Mask R-CNN by 2.6, 17.6, 3.7, 3.0, 2.8, 2.8, 9.4, 12.7, and 14.0 percentage points, respectively. Moreover, SABE-YOLO maintained comparable model parameters and computational complexity to YOLOv8n-seg. Therefore, SABE-YOLO demonstrates superior overall performance and is well suited for efficient segmentation of irregular weld seams.

Although SABE-YOLO achieves a lower FPS than YOLOv8n-seg (127 vs. 217), this is primarily due to the additional computational pathways introduced by the SAFM and C2f-BEAMs for enhanced feature modeling and boundary refinement, which introduce a certain degree of inference latency. However, compared to models such as Mask DINO and Mask2Former, SABE-YOLO maintains a high AP(50–95) of 46.3% while achieving more than six times the inference speed, demonstrating a favorable trade-off between real-time performance and segmentation accuracy.

**Figure 10:**
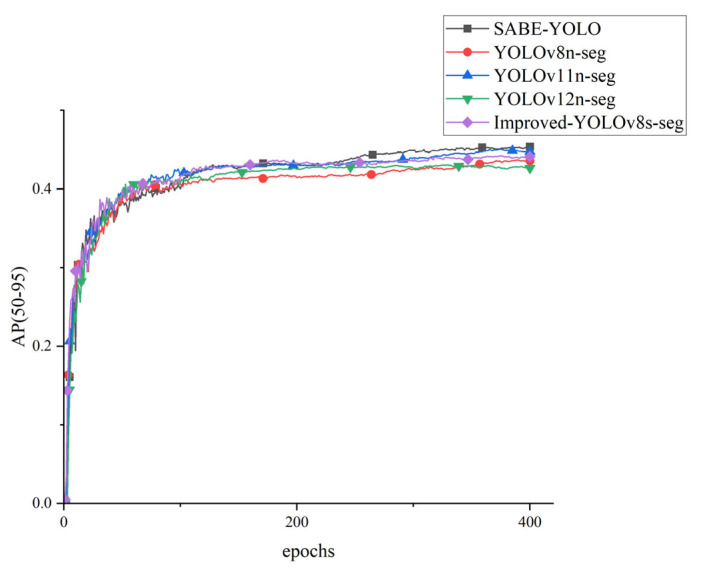
Comparison of AP(50–95) training curves between SABE-YOLO and other YOLO-series instance segmentation models.

As shown in the training curves in Figure 10, the AP(50–95) of all YOLO-based models increases rapidly during the first 100 epochs and gradually stabilizes. Among them, the SABE-YOLO model demonstrates superior optimization characteristics, achieving faster convergence, smoother training curves with lower fluctuations, and ultimately the highest AP(50–95) value. This notable convergence advantage indicates that SABE-YOLO possesses stronger gradient propagation capability and more efficient feature extraction during parameter optimization.

**Figure 11:**
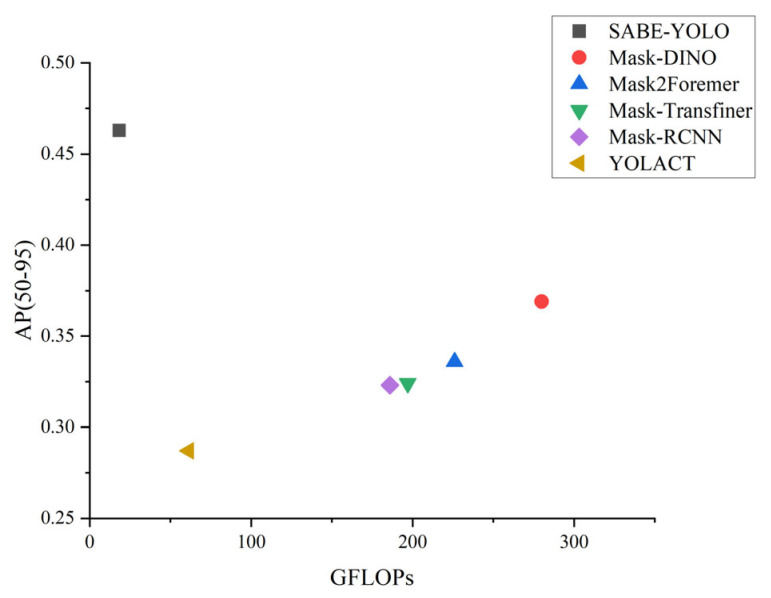
Performance comparison between SABE-YOLO and other mainstream instance segmentation models in terms of GFLOPs and AP(50–95).

As illustrated in Figure 11, compared with other mainstream instance segmentation models, SABE-YOLO achieves higher detection accuracy while maintaining relatively low computational complexity, reflecting a favorable trade-off between performance and efficiency. Experimental results show that SABE-YOLO exhibits enhanced feature extraction and mask prediction capabilities in segmenting weld seams with elongated structures, blurred boundaries, and complex shapes, thereby demonstrating significant advantages in overall performance.

Although SABE-YOLO achieves only a 0.4% improvement in precision over YOLOv8n-seg at the 0.5 IoU threshold, it outperforms YOLOv8n-seg by 3% in AP(50–95). This indicates that SABE-YOLO delivers superior performance under stricter IoU thresholds, demonstrating enhanced accuracy in weld seam localization and better preservation of boundary details.

### 4.4. Qualitative Results

**Figure 12:**
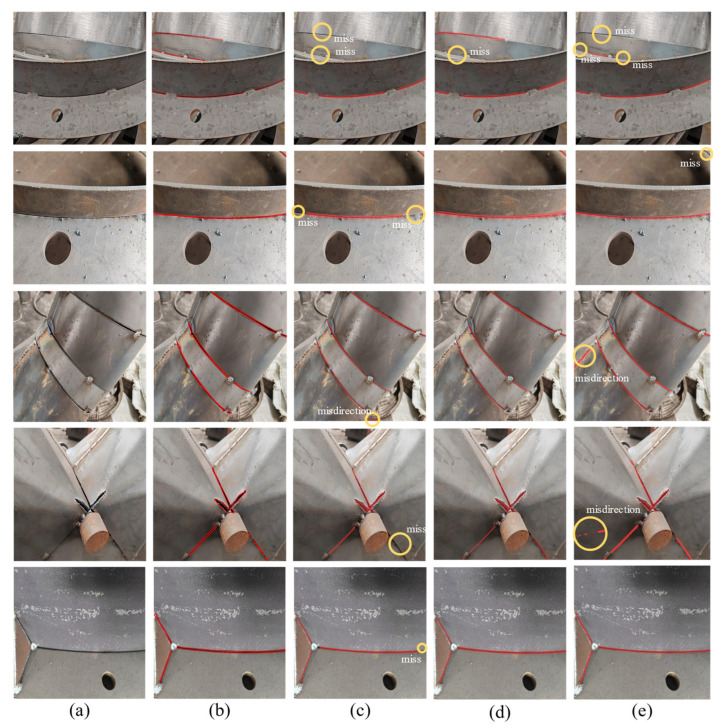
Visual comparison of SABE-YOLO with existing instance segmentation methods. (**a**) Original image, (**b**) ground truth, (**c**) YOLOv8n-seg, (**d**) SABE-YOLO, (**e**) Mask DINO.

To qualitatively evaluate the performance of the SABE-YOLO model in instance segmentation tasks, it was compared with two representative models: the single-stage YOLOv8n-seg and the query-based Mask DINO [10]. Figure 12 presents the segmentation results of weld seam images from the test set using the three models. Notably, the first row showcases failure cases under challenging conditions such as severe occlusion and long viewing distance, aiming to analyze the limitations of each model in complex environments.

From the comparison results, SABE-YOLO successfully segments the weld seam regions in most test images with superior boundary preservation and no apparent false positives or missed detections, except in the failure cases shown in the first row. In contrast, YOLOv8n-seg often exhibits significant omission along weld boundaries and tends to produce false positives, while Mask DINO performs reasonably in maintaining overall structure but still suffers from unstable segmentation masks in cases of blurry or elongated welds.

In the failure cases illustrated in the first row of Figure 12, although SABE-YOLO misses one of the weld seams, it still better preserves the overall structural contours and boundary details compared to the other models.

Overall, by incorporating structure-aware and boundary-enhancing mechanisms, SABE-YOLO demonstrates strong segmentation adaptability and edge retention across various complex scenarios. Its robustness is particularly evident under extreme conditions such as heavy occlusion and distant views. Furthermore, the inclusion of failure cases highlights the need for future improvements in real-world industrial deployments. Strategies such as temporal modeling and 3D perception could be explored to further enhance the model’s practical performance and environmental adaptability.

### 4.5. Ablation Study

To further verify the effectiveness of the proposed improvements in this study, a series of ablation experiments were conducted using the same dataset for training and testing. Based on the YOLOv8n-seg architecture, a more efficient network was constructed by introducing SAFM into the backbone to replace the original C2f module, incorporating C2f-BEAM into the neck, and substituting the original loss function. A total of eight experimental groups were designed to evaluate the contribution of each component individually and in combination. The results of the ablation study are presented in Table 2.

As shown in Table 2, the introduction of C2f-BEAM into the neck network improves the AP(0.5–0.95) of the segmentation model by 0.4 percentage points, indicating that the enhanced module improves the extraction of boundary details and allows more edge features to be retained during feature fusion, thereby strengthening the expression of key features. Incorporating the SAFM multiplication fusion module into the backbone resulted in a 0.9 percentage point improvement in AP(0.5–0.95), indicating that SAFM effectively enhanced the perception of elongated weld structures and enabled improved structural feature extraction. Replacing the CIoU loss function in YOLOv8n-seg with the proposed Inner-MPDIoU results in a 0.6-point increase in AP(0.5–0.95), suggesting that Inner-MPDIoU enhances localization for narrow welds and adapts better to complex, multi-angled weld contours. This not only accelerates model convergence but also improves segmentation accuracy.

The proposed SABE-YOLO model achieves an AP(0.5–0.95) of 46.3%, representing a 3-percentage-point improvement over the original YOLOv8n-seg model. These results demonstrate that, compared to YOLOv8n-seg, SABE-YOLO possesses stronger structure-awareness and boundary extraction capabilities, thereby delivering superior performance in weld seam instance segmentation.

### 4.6. Performance Comparison of Different Loss Functions on YOLOv8n-Seg

To investigate the impact of different loss functions on model performance, we modified the original YOLOv8n-seg model by replacing its original CIoU loss function with MPDIoU, Inner-MPDIoU, and EIoU [40], respectively. Table 3 presents the performance comparison of these four loss functions applied to the YOLOv8n-seg model.

The results in Table 3 demonstrate that when using Inner-MPDIoU as the loss function, precision increased by 1.6%, 0.3%, and 3.0% compared to EIoU, CIoU, and MPDIoU, respectively. Similarly, AP(50–95) improved by 2.7%, 0.6%, and 2.9%, and the F1-score rose by 1.65%, 0.46%, and 1.75%, respectively. Notably, the number of parameters and computational complexity remained unchanged across all settings.

**Figure 13:**
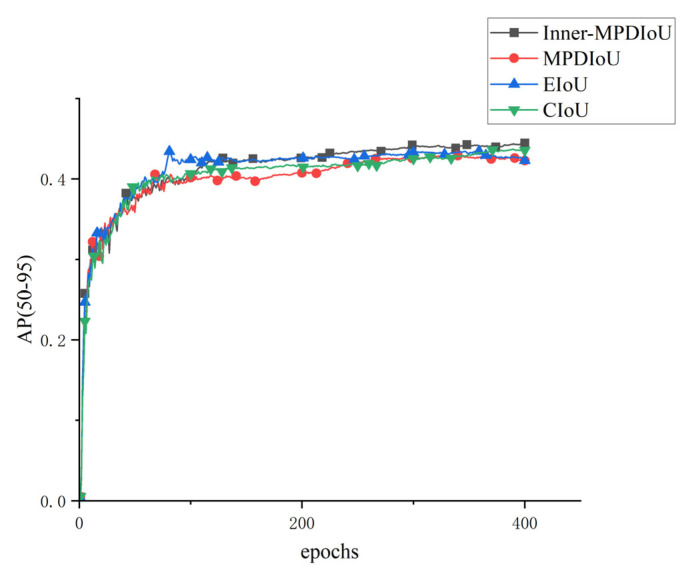
Comparison of AP(50–95) training curves under different loss functions.

As illustrated in Figure 13, the AP(50–95) curves over 400 training epochs indicate that all four loss functions enabled a rapid increase in segmentation accuracy and convergence within 300 epochs. Inner-MPDIoU achieved faster convergence and exceeded the performance of the other loss functions after 220 epochs, maintaining the highest level of accuracy. Although EIoU also converged quickly, it exhibited noticeable oscillations during early training, particularly before 150 epochs. Both MPDIoU and CIoU maintained relatively stable training curves, but their final AP(50–95) values were still lower than those of Inner-MPDIoU. Therefore, Inner-MPDIoU was adopted as the loss function in the SABE-YOLO architecture.

### 4.7. Evaluation of Different Scaling Factors in Inner-MPDIoU

To investigate the impact of different scaling factors in Inner-MPDIoU, comparative experiments were conducted by adjusting the ratio parameter to 0.75, 0.9, 1.0, 1.1, and 1.25 based on the original YOLOv8n-seg model. Specifically, ratios of 0.75 and 0.9 were used to shrink the target area, aiming to evaluate model performance on smaller weld seams or fine-grained features while avoiding excessive focus on redundant regions. A ratio of 1.0 was used to maintain the original target size and was considered as the baseline for comparison. Ratios of 1.1 and 1.25 were used to enlarge the target area, aiming to evaluate adaptability to larger weld seams or complex backgrounds while reducing potential misjudgments at object boundaries. The experimental results are presented in Table 4.

As shown in Table 4, the best performance was achieved when the scaling factor was set to 0.9, with both precision and AP(50–95) significantly outperforming other experimental groups. This indicates that a moderate reduction in the bounding box size can effectively enhance segmentation accuracy by improving attention to the target region.

Specifically, when the scaling factor was greater than or equal to 1, the bounding box became too large and included too much irrelevant background. This increased noise and reduced the sensitivity of the loss function to the core region, resulting in blurred segmentation results. When the ratio was set to 0.75, the bounding box became overly narrow and caused a loss of edge information, making it difficult to capture irregular contours. In contrast, a ratio of 0.9 allowed the bounding box to fully cover the main weld seam while excluding about 10 percent of external background noise. This helped improve confidence in the core region. Based on these findings, a scaling factor of 0.9 was adopted in Inner-MPDIoU to achieve optimal segmentation performance.

### 4.8. Selection of Dual-Branch Fusion Strategy in SAFM

To investigate the impact of different dual-branch fusion strategies within the Structure-Aware Fusion Module (SAFM) on model performance, we designed and compared three fusion methods. All experiments were conducted based on the original YOLOv8n-seg architecture under identical backbone and training configurations, aiming to assess their effect on weld seam instance segmentation.

**Figure 14:**
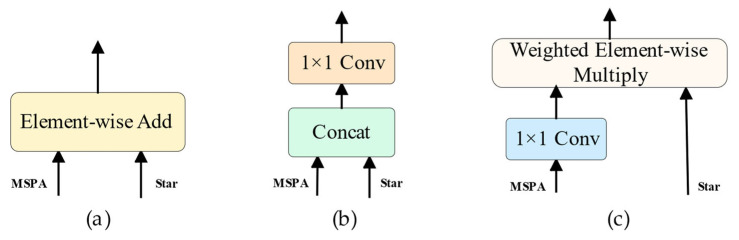
Comparison of different fusion strategies in SAFM. (**a**) Element-wise Add: Feature maps from both branches are combined via direct element-wise summation. (**b**) Concatenation + Convolution: Features are concatenated along the channel dimension and passed through a 1 × 1 convolution to reduce dimensionality. (**c**) Weighted Element-wise Multiply: The MSPA branch generates a weight map through a 1 × 1 convolution, which is then multiplied element-wise with the Star branch to dynamically emphasize structural cues.

In SAFM, a dual-branch structure is adopted: one branch incorporates strip pooling attention (MSPA), while the other (Star branch) captures complementary spatial information. We implemented the following three fusion strategies, as illustrated in Figure 14.

To evaluate the effect of each strategy, we conducted ablation experiments using the same training setup. The results are shown in Table 5.

It was observed that the Weighted Element-wise Multiply approach achieved the best balance between segmentation accuracy and computational efficiency. Essentially, this method serves as a lightweight attention mechanism that dynamically suppresses redundant information and enhances geometric structure representation by modulating the Star branch with learned weights from the MSPA path. This design significantly improves the ability pf the model to capture the directional characteristics of slender welds.

In summary, we adopt Weighted Element-wise Multiply as the default fusion strategy in SAFM to ensure optimal trade-off between performance and efficiency.

### 4.9. Effect of the Number of Boundary Enhancement Paths in BEAM on Performance

In C2f-BEAM, the BEAM serves as the core substructure, responsible for fusing multi-scale semantic and boundary information. It consists of multiple Boundary Enhancement Paths, each composed of average pooling, convolutional layers, and boundary enhancement submodules, forming a hierarchical structure through cascaded stacking.

To explore the impact of the number of enhancement paths in the BEAM on segmentation performance, this study conducted comparative experiments by varying the number of stacked boundary paths *d*, constructing BEAMs with two to four layers. These were integrated into the baseline YOLOv8n-seg architecture, with all other components kept unchanged. The performance in terms of accuracy, boundary awareness, and inference speed was evaluated. Results are shown in Table 6.

As shown in Table 6, increasing the number of enhancement paths from 2 to 3 significantly improves overall segmentation performance (AP(50–95)), indicating that multi-layer paths help in modeling boundary details at various scales. However, further increasing the path count to four leads to saturated or slightly decreased performance, possibly due to feature redundancy causing information confusion. Additionally, more paths increase the model size and computational cost, resulting in slower inference. Therefore, the BEAM adopts three boundary enhancement paths by default to strike an optimal balance between accuracy and efficiency.

### 4.10. Zero-Shot Generalization Experiment

To further evaluate the structure-aware and boundary modeling capabilities of the proposed SABE-YOLO model under cross-domain conditions, a zero-shot inference generalization experiment was conducted on 100 images selected from the test and validation sets of the Crack Segmentation Dataset. In this experiment, the model performs inference directly using weights trained solely on the self-built weld seam dataset, without any additional transfer learning or fine-tuning.

Although cracks and weld seams differ in semantic category and application context, they share common visual characteristics such as elongated structures, ambiguous boundaries, and weak texture features, all of which pose similar challenges for structure modeling. Therefore, this task serves as a reasonable benchmark for testing the structural generalization ability of the model.

Three representative instance segmentation models were selected for comparison: YOLOv8n-seg, YOLOv12n-seg, and Mask DINO. None of the models were trained or fine-tuned on the Crack Segmentation Dataset, ensuring consistency in the zero-shot inference setting. The results are presented in Table 7.

Despite being trained on a domain different from the crack dataset, SABE-YOLO achieves the highest AP(50–95) score of 34.3%, outperforming other mainstream models and validating the excellent cross-domain generalization capabilities of the SAFM and C2f-BEAMs.

Notably, all models obtain AP(50–95) scores below 35% on the Crack Segmentation Dataset, highlighting the considerable domain shift inherent in structural segmentation tasks. To address this challenge, future research may explore style alignment, structural self-supervision, or cross-domain fine-tuning to further enhance model robustness and generalization in heterogeneous scenarios.

## 5. Discussion

The proposed SABE-YOLO method outperforms existing mainstream instance segmentation approaches across multiple performance metrics. It demonstrates superior feature modeling and segmentation capabilities, particularly in industrial scenarios involving slender weld structures, blurred boundaries, and complex shapes. Experimental results show that the AP(50–95) of SABE-YOLO reaches 46.3%, representing a 3% improvement over YOLOv8n-seg, while maintaining similar parameter count and computational complexity. This reflects a favorable balance between performance and efficiency.

The performance improvement of SABE-YOLO is primarily attributed to three key enhancements. First, SAFM incorporates a strip pooling attention branch and a Star branch to efficiently capture the slender geometry of weld seams, enhancing the representation of both local directional and global structural features. Second, C2f-BEAM extracts and integrates multi-scale boundary details, guiding the network to focus on weld boundaries and improving boundary preservation and pixel-level mask prediction. This module notably reduces false positives and missed detections, especially near weld endpoints or in blurred regions. Third, the Inner-MPDIoU loss function addresses geometric constraints by applying center region scaling and minimizing corner point distance, which alleviates the gradient instability observed with CIoU in slender structures. This leads to a smoother optimization process and improved localization robustness. Ablation experiments further confirm that each of these modules contributes independently and synergistically to overall performance improvement within the framework.

In comparative experiments with several representative instance segmentation models, including YOLOv8n-seg, Mask2Former, and Mask DINO, the proposed SABE-YOLO model not only achieved superior AP(50–95) performance but also maintained low computational complexity (18.3 GFLOPs), a compact parameter size (6.6M), and a high inference speed of 127 FPS. This significantly exceeded the speed of Transformer-based two-stage methods, such as Mask DINO (15 FPS), demonstrating strong potential for deployment on edge devices, particularly in resource-constrained welding scenarios. Further analysis of the Inner-MPDIoU loss function revealed that moderately shrinking the predicted bounding boxes to the core regions of targets (ratio = 0.9) effectively suppressed background noise and improved focus of attention, contributing to enhanced segmentation accuracy. Additionally, experimental results confirmed that applying weighted element-wise multiplication in the dual-branch fusion strategy of SAFM and setting the number of boundary enhancement paths in BEAM to three yielded optimal performance.

To assess cross-domain generalization capability, zero-shot inference was performed using 100 crack images selected from the test and validation sets of the Crack Segmentation Dataset. Despite the semantic differences between welds and cracks, both share similar visual characteristics, such as elongated shapes, blurred boundaries, and weak textures. Under this challenging setting, SABE-YOLO achieved the highest AP(50–95) score of 34.3%, significantly outperforming the baseline models, validating the effectiveness of the structure-aware module (SAFM) and boundary-enhancement mechanism (C2f-BEAM) in transferring to visually analogous domains. These findings suggest the potential of the model to generalize to other slender-structure detection tasks such as crack localization and linear feature extraction.

In parallel, recent advancements in robotic perception and manipulation emphasize the importance of generalization and adaptability in vision models. Bayraktar et al. [41] proposed a hybrid image dataset framework to bridge the domain gap between simulated and real-world environments, thereby improving robustness in structurally complex scenarios. Their team also developed a robotic manipulation system [42] that integrates deep semantic understanding and payload estimation, illustrating the synergy between structure perception, task planning, and action execution. These efforts align closely with the goals of this study, further demonstrating the practical value of combining structure-aware modeling with semantic guidance in industrial vision tasks.

From an application perspective, the lightweight design and relatively low computational burden make the proposed model well-suited for deployment on edge devices such as the NVIDIA Jetson series, meeting the requirements of industrial welding robots for real-time processing and operational reliability. However, the current model is based on 2D imagery and lacks explicit depth modeling, which limits adaptability in complex 3D welding environments.

To address this limitation, future work may explore the integration of multimodal sensory inputs, such as depth cameras, stereo vision, or structured light, to improve spatial modeling. The model could also be extended by incorporating point cloud processing techniques to better capture geometric details. Moreover, strategies such as model pruning and knowledge distillation may be employed to enhance inference efficiency. Dynamic feature selection methods like Conditional Pooling [43] could further improve computational performance, offering promising directions for lightweight segmentation model design.

In conclusion, SABE-YOLO significantly enhances weld seam instance segmentation performance through structure-aware and boundary modeling mechanisms, while maintaining high efficiency. The model demonstrates strong industrial applicability and scalability. Future research will further explore 3D modeling, multimodal perception, and embedded deployment to advance the practical implementation of intelligent weld seam recognition in industrial manufacturing scenarios.

## 6. Conclusions

This study addresses the challenges of locating slender structures and preserving weak boundaries in weld seam instance segmentation by proposing an instance segmentation model, SABE-YOLO, which integrates structure-aware and boundary-enhanced mechanisms. First, SAFM is introduced into the backbone network, employing a multi-head strip self-attention mechanism and cross-subspace feature multiplication strategy to significantly enhance the perception of slender weld structures. Second, C2f-BEAM is designed in the neck network, combining multi-scale boundary extraction, adaptive spatial feature aggregation, and the EMA mechanism to effectively improve segmentation performance on weak boundary details. Finally, the Inner-MPDIoU loss function is introduced to focus on the internal region of the target and constrain the corner positions, thereby improving robustness and accuracy in object localization and angle estimation.

In practical evaluations, SABE-YOLO achieved an accuracy of 89.0%, a recall of 83.6%, and an AP(50–95) of 46.3%, outperforming mainstream models such as YOLOv12n-seg, Mask-DINO, and Mask2Former across multiple metrics. The results demonstrate a well-balanced trade-off between overall performance and computational efficiency. Furthermore, visual experiments confirmed strong segmentation integrity and effective boundary preservation under complex weld seam geometries.

Overall, SABE-YOLO provides an effective technical solution for high-precision and low-complexity visual perception in pre-scanning welding tasks, demonstrating strong potential for engineering deployment. Future work will focus on enhancing capability in 3D information understanding and temporal modeling, such as integrating laser point cloud data and introducing spatiotemporal attention mechanisms, to further improve generalization and robustness in real-world industrial scenarios.

## Figures and Tables

**Table 1:** Performance comparison of SABE-YOLO with mainstream instance segmentation models on the test dataset.

Model	P%	R%	AP(50–95)%	Params/M	GFLOPs	FPS
Improved YOLOv8s-seg [28]	89.3	83.2	43.7	6.36	21.2	159
YOLOv8n-seg [12]	88.6	83.8	43.3	3.3	12	217
YOLOv11n-seg [23]	89.9	82.9	43.5	2.8	10.2	231
YOLOv12n-seg [24]	90.2	83.7	43.5	2.8	10.2	224
YOLACT [6]	-	-	28.7	34.7	61.4	86
Mask-RCNN [5]	-	-	32.3	44	186	48
Mask DINO [10]	-	-	36.9	52	280	15
Mask Transfiner [39]	-	-	32.4	44	197	18
Mask2Former [11]	-	-	33.6	44	226	6
SABE-YOLO (ours)	89	83.6	46.3	6.6	18.3	127

**Table 2:** Ablation study results of the SABE-YOLO network on the test set. A check mark (√) indicates that the corresponding module is enabled.

YOLOv8n-seg	C2f-BEAM	SAFM	Inner-MPDIoU	AP(50–95)%	Params/M	GFLOPs
√				43.3	3.2	12.1
√	√			43.7	3.3	12.3
√		√		44.2	6.5	18.3
√			√	43.9	3.2	12.1
√	√	√		45.5	6.6	18.4
√	√		√	44.6	3.3	12.3
√		√	√	45.0	6.5	18.3
√	√	√	√	46.3	6.6	18.4

**Table 3:** Comparison of segmentation results using different loss functions.

Loss Function	P%	R%	AP(50–95)%	F1-Score%	Params/M	GFLOPs
EIoU [40]	87.3	82.7	41.2	84.94	3	12
CIoU [37]	88.6	83.8	43.3	86.13	3	12
MPDIoU [18]	85.9	83.8	41	84.84	3	12
Inner-MPDIoU	88.9	84.4	43.9	86.59	3	12

**Table 4:** Comparison of segmentation performance using different scaling factors in Inner-MPDIoU.

Ratio	P%	R%	AP(50–95)%	F1-Score%	Params/M	GFLOPs
0.75	86.9	85	41.9	85.94	3.258	12
0.9	88.9	84.4	43.9	86.59	3.258	12
1	85.9	83.8	41	84.84	3.258	12
1.1	87.8	83.6	42.6	85.65	3.258	12
1.25	86.8	84.3	42.6	85.53	3.258	12

**Table 5:** Comparison of segmentation results using different fusion strategies.

Fusion Strategy	AP(50–95)%	Params/M	GFLOPs	FPS
Element-wise Add	42.1	6.45	18.2	129
Concat + Conv	42.8	6.88	19.1	127
Weighted Multiply	44.2	6.5	18.3	127

**Table 6:** Comparison of segmentation performance using different numbers of enhancement paths.

Number of Enhancement Paths (*d*)	AP(50–95)%	Params/M	GFLOPs	FPS
2	42.6	3.33	12.1	213
3	43.7	3.34	12.1	207
4	42	3.35	12.2	200

**Table 7:** Zero-shot inference results on the Crack Segmentation Dataset.

Model	AP(50–95)%	Params/M	GFLOPs	FPS
YOLOv8n-seg	14	3.3	12	97
YOLOv12n-seg	21.1	2.8	10.2	99
Mask DINO	19.2	52	280	11
SABE-YOLO (ours)	34.3	6.6	18.3	70

## Data Availability

Data are contained within the article.
